# Down-Regulation of Toll-Like Receptor 5 (TLR5) Increased VEGFR Expression in Triple Negative Breast Cancer (TNBC) Based on Radionuclide Imaging

**DOI:** 10.3389/fonc.2021.708047

**Published:** 2021-07-15

**Authors:** Wen Jiang, Yeming Han, Ting Liang, Chao Zhang, Feng Gao, Guihua Hou

**Affiliations:** ^1^ Key Laboratory for Experimental Teratology of the Ministry of Education and Research Center for Experimental Nuclear Medicine, School of Basic Medical Sciences, Shandong University, Jinan, China; ^2^ Radiology Department, Qilu Hospital of Shandong University, Jinan, China

**Keywords:** toll-like receptor 5, vascular endothelial growth factor receptor, triple negative breast cancer, radioiodine 125, autoradiography

## Abstract

In this study, GFP-tagged TNBC 4T1 cells with down-regulated TLR5 expression (TLR5^−^ 4T1) and normal TLR5 expression (TLR5^+^ 4T1) were constructed, respectively. RT-PCR and Western blot studies showed that down-regulation of TLR5 obviously increased the expression of VEGFR in 4T1 cells. Highly stable radio-probes ^125^I-anti-TLR5 mAb/^125^I-VEGF/^125^I-IgG were obtained with labeling rates over 85% and radiochemical purities above 90%. Among these three probes, ^125^I−anti−TLR5 mAb and ^125^I-VEGF were used for specifically imaging TNBC, while ^125^I-IgG was used for comparison. Whole-body phosphorus autoradiography showed clear imaging at 48 h after injection of ^125^I-anti-TLR5 mAb and ^125^I-VEGF also provided clear imaging at 24 h. Biodistribution study demonstrated a higher tumor uptake of ^125^I-anti-TLR5 mAb in TLR5^+^ group compared with that in TLR5^−^ group (P < 0.05), whereas tumor uptake of ^125^I-VEGF in TLR5^+^ group was lower than that in the TLR5^−^ group (P < 0.05). Immunohistochemical staining suggested that the expression of TLR5 was lower, whereas the expression of VEGFR, CD31, and MVD (microvessel density) was higher in TLR5^−^ tumor-bearing mice. In summary, the down-regulation of TLR5 in TNBC promoted the VEGFR expression and angiogenesis, resulting in the proliferation of TNBC cells. TLR5/VEGF might be a better indicator for monitoring the development of TNBC.

## Introduction

Breast cancer is the most common malignant tumor in women, and TNBC accounts for about 15% of breast cancer (1). Compared with non-TNBC, TNBC is reported with high metastasis and recurrence rates, as well as poor prognosis, and there is no effective targeted treatment for TNBC (2). Until now, chemotherapy is still the only systemic therapy in clinics (3, 4), and the treatment effect is not ideal when it is in the advanced stage. Therefore, it is of great significance and importance to search for novel targets for early diagnosis of TNBC with high selectivity and to clarify the underlying mechanism.

Angiogenesis is a promising target for TNBC, and vascular endothelial growth factor (VEGF) has been considered as an important angiogenic factor at present (5). VEGFR is the main receptor of VEGF, and its expression in TNBC is found higher than that in non-TNBC (6–8). With the increase of tumor volume, the expression of VEGFR is also significantly increased, so VEGFR could be taken as a specific marker of TNBC. There is a unique autocrine signal loop in the surface of the tumor cells, which makes breast cancer cells promote angiogenesis by the activation of VEGFR, providing further nutrition for tumor growth (9, 10). More importantly, angiogenesis inhibitors, such as bevacizumab and sorafenib, showed relative therapeutic effects for TNBC in clinics (11).

Molecular imaging, which combines advanced imaging technology with molecular biology, can be defined as an imaging mean to detect physiological or pathological processes non-invasively at the cellular and molecular levels (12). Compared with traditional imaging, molecular imaging can qualitatively or quantitatively provide molecular information about diseases before anatomical changes, which not only improves the specificity, sensitivity, and accuracy of diagnosis but also effectively improves the rate of early diagnosis so as to achieve “early detection, early diagnosis, and early treatment.” The biomarkers used for molecular imaging include peptides, antibody, and antibody fragments (13), which allow to qualitatively and quantitatively trace biological behaviors. Compared with other imaging methods, nuclear molecular imaging can obtain more sensitive *in vivo* images using radiolabeled probes, which plays an important role in basic research and clinical diagnosis (14). Nuclear molecular imaging is the most advance technique used in clinics with a higher sensitivity for diagnosis of some diseases, such as inflammation, tumor, cardiovascular, and so on. In inflammation, such as traumatic osteomyelitis, nuclear imaging can diagnose traumatic osteomyelitis timely and accurately, whereas X-ray and CT cannot distinguish infection from inflammation (15). In tumors, especially in malignant tumors, molecular changes lead to up-regulation or down-regulation of molecular targets (16). These targets can be used for imaging and treatment. For example, in the diagnosis of paraganglioma, ^68^Ga-labeled somatostatin analogs simplified the imaging method of paraganglioma and described the characteristics of the tumor at the cellular and molecular level (17). ^18^F-FDG, the most commonly used radiotracer in clinical tumor diagnosis, plays an important role in tumor diagnosis, staging, curative effect evaluation, prognosis prediction, and so on (18). However, ^18^F-FDG uptake is related to cellular glucose metabolism, and high ^18^F-FDG uptake is consistent with cellular high metabolism, so high ^18^F-FDG uptake not only exists in tumors but also happens in infectious and non-infectious inflammation (19, 20). ^18^F-FDG is lack of specificity in tumor diagnosis, making false-positive or false-negative misdiagnosis sometimes. Hence, it is urgent to find more specific molecular targets for precise diagnosis of tumor by nuclear molecular imaging.

It has been reported that TLR5 was closely related to the proliferation, invasion, and metastasis of TNBC. Down-regulation of TLR5 enhanced proliferation, invasion, and metastasis of TNBC (19, 21). The TNBC-bearing mice model showed similar biological behavior to human diseases (22). In our previous study, we found that TLR5-targeted radio-probe could not image TNBC clearly when TLR5 was down-regulated in TNBC (23), so it is necessary to find a novel target to monitor the development of TNBC. More importantly, whether the down-regulation of TLR5 in TNBC could affect the expression of VEGFR and angiogenesis is still unclear. In this study, the radio-probes targeting TLR5/VEGF were prepared to investigate the influence of down-regulation of TLR5 on VEGFR expression and angiogenesis, and TLR5/VEGF might be a potential novel indicator of TNBC.

## Materials and Methods

### Cell Culture

TNBC cell line 4T1 was purchased from the American Type Culture Collection (ATCC) and preserved by Center for Experimental Nuclear Medicine of Shandong University. The cells were cultured in RPMI-1640 medium supplemented with 10% fetal bovine serum (FBS; Sigma) and 1% penicillin/streptomycin (Hyclone) at 37°C in an incubator with 5% CO_2_ and saturated humidity.

### Lentivirus Transfection and Screening

4T1 cells were plated in 96-well plate overnight. Lentivirus-shRNA-TLR5 together with negative Lentivirus (GenePharma) was added to the culture medium with a MOI value of 10. After incubation for 48 h, 20 μg/ml puromycin (Selleck) was added and the cells were screened continuously for 7 days. Finally, bright field and the corresponding fluorescence field were captured with microscope, and the transfection efficiency was calculated.

### Expression of TLR5 and VEGFR mRNA

The expression of TLR5/VEGFR mRNA was detected by semi-quantitative reverse transcription (RT)-PCR. Total RNA from 4T1 cells was extracted using TRIzol ^®^reagent (Invitrogen; Thermo Fisher Scientific, Inc.). The first strand cDNA was synthesized using TransScrip^®^ First-Strand cDNA Synthesis SuperMix Kit (Beijing full Gold Biology Co., Ltd.). According to the instructions of EasyTaq PCR kit (Beijing full Golden Biology Co., Ltd.), the PCR amplification was performed with the procedure as follows: pre-denaturation at 94°C for 5 min, followed by 33 cycles of denaturation at 94°C for 30 s, annealing at 58°C for 30 s, extension at 72°C for 30 s, and a final extension at 72°C for 10 min. The PCR primers used as follows: TLR5 forward, 5′-GCAGGATCATGGCATGTCAAC-3′ and reverse, 5′-ATCTGGGTGAGGTTACAGCCT-3′; VEGFR forward, 5′-TTTGGCAAATACAACCCTTCAGA-3′ and reverse, 5′-GCTCCAGTATCATTTCCAACCA-3′; GAPDH forward, 5′-GGAGCGAGATCCCTCCAAAAT-3′ and reverse, 5′-GGCTGTTGTCATACTTCTCTCATGG-3′. The PCR products were electrophoresis in agarose gels and then scanned on UV analyzer.

### Expression of TLR5 and VEGFR Protein

Total protein was extracted from 4T1 cells using RIPA lysis buffer (Servicebio) supplemented with phenylmethysulfonyl fluoride (PMSF, 1:100), protease inhibitor cocktail (1:100), and phosphatase inhibitor cocktail (1:50) for 30 min on ice. According to the instructions of PAEG gel rapid preparation kit (Epizyme), 10% mini glue was prepared. Then, 20 µg of total protein was loaded onto the corresponding PAEG gel well along with Chameleon Duo ladder protein marker (Epizyme). The electrophoresis conditions were as follows: the constant voltage of concentrated gel and separation gel was 80 and 120 mV, respectively. Then the 0.45μm PVDF membrane (Merck) was activated by methanol, and the protein was transferred to PVDF membrane with 200 mA for 90 min. The membrane was blocked in 5% (w/v) skimmed milk blocking buffer for 2 h at room temperature and washed with TBS containing Tween-20 (TBST) for 30 min, and then incubated with the primary antibody overnight at 4°C. The primary antibody used was as follows: TLR5 Rabbit mAb (1:500) (Abways); VEGFR Rabbit mAb (1:1000) (CST); GAPDH Rabbit pAb (1:1000) (Abcam). The membranes were washed with TBST for 30 min and incubated with secondary antibody (Abways) for 2 h at room temperature. Finally, the membranes were scanned and quantitatively analyzed by TANON 4200 imaging system and Image J software.

### Animal Model

Female BABL/c mice, aged 6 to 8 weeks and weighted 18 to 21 g, were provided by Beijing Vital River Laboratory Animal Technology Co., Ltd. TLR5^+^ and TLR5^−^ 4T1 cells were digested by 0.25% trypsin and suspended in phosphate-buffered saline (PBS). Each mouse was subcutaneously injected with 0.2 ml cells (1 × 10^7^/ml) on the right shoulder. Mice were fed routinely, and the tumor size and growth status of mice were monitored every day. When the diameter of the tumor reached 5 to 10 mm, the experiment could be carried out.

### Preparation of the ^125^I-anti-TLR5 mAb/^125^I-VEGF/^125^I-IgG

Iodogen method was used for labeling anti-TLR5 mAb (Abways), VEGF (PeproTech), and IgG (Solarbio) with ^125^I according to the reference (24). Labeling yield was obtained by dividing the activity of the protein sample to that of the free ^125^I sample. Radiochemical purity and stability in normal saline (NS) and fetal bovine serum (FBS) were confirmed with paper chromatography.

### Dynamic Whole-Body Phosphor-Autoradiography With ^125^I-anti-TLR5 mAb and ^125^I-IgG

One day before injection of radiotracers, mice were fed with 3.5% sodium iodide solution to block the thyroid gland uptake of iodine. The tumor-bearing mice were intraperitoneally injected with ^125^I-anti-TLR5 mAb or ^125^I-IgG (^125^I-IgG used as a non-specific control). The whole-body dynamic phosphor-autoradiography was performed at 24, 48, and 72 h after injection. Mice were anesthetized by injecting intraperitoneally with 0.6% pentobarbital sodium (0.1 ml/10 g). The anesthetized mice were facing up, and the back was close to the phosphor screen plate for 20 min. Later, the plate was quickly put into the Cyclone Plus scanner (PerkinElmer Life Sciences) for image acquisition. Semi-quantitative analysis was performed by manually drawing rectangular regions of interest within the target area. Digital light units per square millimeter (DLU/mm^2^) measurements were obtained using OptiQuant™ Image Analysis Software (PerkinElmer Life Sciences). After whole-body phosphor-autoradiography, the tumor was removed out and ex vivo imaging of the tumor was also performed.

### Dynamic Whole-Body Phosphor Autoradiography With ^125^I-VEGF and Blocking Study

The procedure about blocking the uptake of iodine in thyroid gland was the same as above. The tumor-bearing mice were divided into two groups (blocking group and non-blocking group). For blocking group, unlabeled anti-VEGFR pAb (100 μg) (Bioss) was injected 1 h before ^125^I-VEGF injection. The dynamic whole whole-body phosphor-autoradiography was performed at 6, 12, 24, and 48 h after injection of ^125^I-VEGF. The image acquisition method and analysis methods were the same as described above. Tumor was also isolated after whole-body phosphor-autoradiography to perform ex vivo tumor imaging.

### *Ex Vivo* Biodistribution Studies

^125^I-anti-TLR5 mAb, ^125^I-VEGF, and ^125^I-IgG were injected into model mice, respectively. For those mice injected with ^125^I-anti-TLR5 mAb/^125^I-IgG, biodistribution study was performed at 48 h post-injection, whereas for mice injected with ^125^I-VEGF, biodistribution study was performed at 24 h post-injection. The mice were executed, and tumors, blood as well as major tissues/organs of interest (bone, muscle, thyroid, liver, spleen, kidney, small intestine, lung, and heart) were isolated, weighed, and measured with Gamma counter. Tissue radioactivity was expressed as the percent injected dose per gram tissue (%ID/g). The T/NT ratio (target to non-target) was defined as the ratio of radioactivity in the tumor to that in the opposite muscle tissue.

### Fluorescence Imaging

Both 4T1 TLR5^+^/^−^ cells transfected with lentivirus expressed Green Fluorescent Protein (GFP). To avoid fluorescence quenching, the mouse tumor was placed on the imaging plate of the IVIS^®^ Spectrum *in vivo* imaging system (PerkinElmer) for fluorescence imaging immediately it was removed out.

### H&E and Immunohistochemical Staining

Tumors were isolated from both 4T1 TLR5^+^ and TLR5^−^ tumor bearing mice at day 10, fixed and preserved with paraformaldehyde, and then embedded in paraffin to make sections. Immunohistochemical staining with anti-TLR5 pAb (Bioss), anti-VEGFR mAb, and anti-CD31 mAb were performed according to the kit instruction. The slides were observed at a magnification of 100× and 400×. The IOD and corresponding area counting were analyzed(five fields per slides) by the Image-Pro Plus software. In addition, the number of blood vessels marked by CD31 positive staining was also counted.

### Statistical Analysis

All experiments were repeated three times at least, and the data were expressed as mean ± standard deviation. Student’s *t*-test was used by GraphPad Prism software (version 5.01, GraphPad Software, Inc). P < 0.05 was considered a statistically significant difference.

## Results

### Lentivirus Transfection Efficiency and Expression of TLR5 and VEGFR in 4T1 Cells

**Figure 1:**
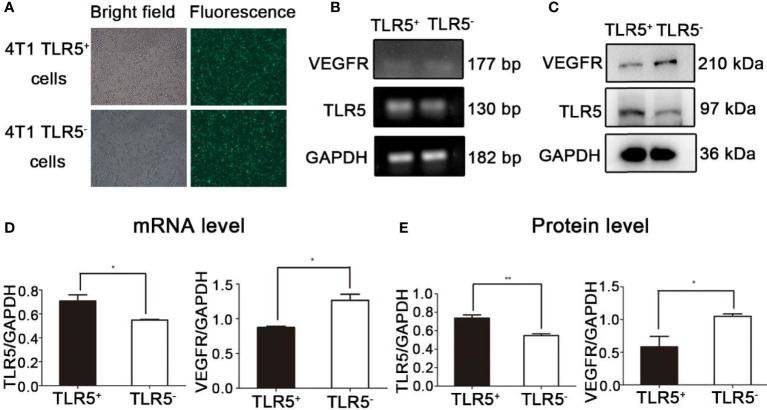
Lentivirus-shRNA-TLR5 and negative lentivirus transfection efficiency in 4T1 cells **(A)**. The TLR5 and VEGFR mRNA expression in TLR5^+^ and TLR5^‐^4T1 cells (n = 3, *P < 0.05) **(B, D)**. The TLR5 and VEGFR protein expression in TLR5^+^ and TLR5^−^ 4T1 cells (n = 3, *P < 0.05, **P < 0.01) **(C, E)**.

As shown in **Figure 1A**, the lentivirus transfection efficiency for 4T1 cells was almost 100%. Compared with the 4T1 TLR5^+^ cells, the expression of TLR5 mRNA and protein was obviously decreased (P < 0.05), whereas the expression of VEGFR mRNA and protein was apparently increased in 4T1 TLR5^−^ cells (P < 0.05, **Figure 1**), which indicated that TLR5 down-regulation apparently increased the expression of VEGFR in 4T1 cells.

### Preparation of ^125^I-anti-TLR5 mAb/^125^I-VEGF/^125^I-IgG

**Figure 2:**
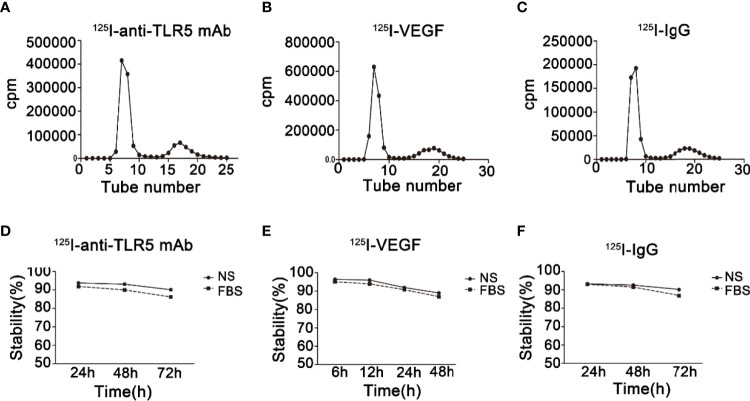
Radiochemical purity and stability of radio-probes. Radiochemical purity of ^125^I-anti-TLR5 mAb/^125^I-VEGF/^125^I-IgG **(A–C)**. Stability of ^125^I-anti-TLR5 mAb/^125^I-VEGF/^125^I-IgG in saline and serum at different time points **(D–F)**.

The labeling rate of ^125^I-anti-TLR5 mAb/^125^I-VEGF/^125^I-IgG was 92.5%, 94.3%, and 89%, respectively (**Figures 2A–C**). The radiochemical purity of ^125^I-anti-TLR5 mAb/^125^I-VEGF/^125^I-IgG was 97.5%, 94.2%, and 97.1%, respectively. These three probes were relatively stable in both saline and serum even for 72 h (**Figures 2D–F**).

### Dynamic Whole-Body Phosphor-Autoradiography With ^125^I-anti-TLR5 mAb/^125^I-IgG and Fluorescence Imaging

**Figure 3:**
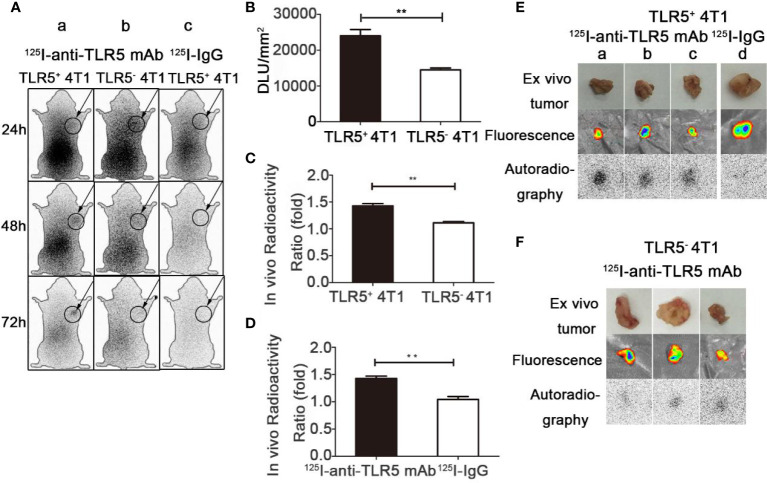
Whole-body phosphor-autoradiography and fluorescence imaging. Whole body phosphor-autoradiography of ^125^I-anti-TLR5 mAb in TLR5^+^ 4T1 tumor bearing mice **(A**a**)** and TLR5^−^ 4T1 tumor bearing mice **(A**b**)** at 24, 48, and 72 h post-injection, and whole-body phosphor-autoradiography images of ^125^I-IgG **(A**c**)** in TLR5^+^ 4T1 tumor bearing mice at 24, 48, and 7 2h post-injection. DLU/mm^2^ of TLR5^+^ and TLR5^‐^4T1 tumors **(B)**. Comparison of *in vivo* radioactivity ratio (the ratio of DLU/mm2 from tumor and opposite muscle) of TLR5^+^/^−^ 4T1 tumors in phosphor-autoradiography at 48 h **(C)**. Comparison of *in vivo* radioactivity ratio of TLR5^+^ 4T1 tumors between ^125^I-anti-TLR5 mAb and ^125^I-IgG **(D)**. Phosphor-autoradiography and fluorescence imaging of isolated TLR5^+^ 4T1 tumors in ^125^I-anti-TLR5 mAb group **(E**a–c**)**, and ^125^I-IgG group **(E**d**)**. Phosphor-autoradiography and fluorescence imaging of isolated TLR5^−^ 4T1 tumor in ^125^I-anti-TLR5 mAb group **(F)**. The data were presented from three independent experiments and analyzed by Student’s t test. **P < 0.01.

Whole-body phosphor-autoradiography was performed at 24, 48, and 72 h after injection of the ^125^I-anti-TLR5 mAb or ^125^I-IgG. The results were shown in **Figure 3**. The radioactivity uptake of ^125^I-anti-TLR5 mAb in the tumor was much higher at 48 h than that at 24 h in both 4T1 TLR5^+^ and TLR5^−^ tumors. Compared with 4T1 TLR5^−^ tumors, higher radioactivity uptake was detected in 4T1 TLR5^+^ tumor at all checked time points (**Figure 3A**). Semi-quantitative analysis of DLU/mm^2^ about the tumor radioactivity showed 23982.9 for 4T1 TLR5^+^ tumor, whereas only 14482.9 for TLR5^−^ tumor (P < 0.01, **Figure 3B**). The T/NT ratio (tumor/opposite muscle) was 1.4681 and 1.11254 for TLR5^+^ and TLR5^−^ tumor, respectively (*P* < 0.01, **Figure 3C**). There was no obvious radioactivity accumulation in the tumor in ^125^I-IgG group at all checked time points, suggesting that there was a specific accumulation of ^125^I-anti-TLR5 mAb in the 4T1 tumor (P < 0.01, **Figures 3A**c, **D**). For *ex vivo* imaging of isolated tumors, 4T1 TLR5^+^ tumors (**Figure 3E**) showed much higher radioactivity accumulation than 4T1 TLR5^−^ tumors (**Figure 3F**). Moreover, both 4T1 TLR5^+^ and 4T1 TLR5^−^ tumors displayed obvious fluorescence signals (**Figures 3E, F** **)**, which indicated that fluorescence tag could be used for co-location of 4T1 tumor.

### Dynamic Whole-Body Phosphor-Autoradiography With ^125^I-VEGF and Fluorescence Imaging

**Figure 4:**
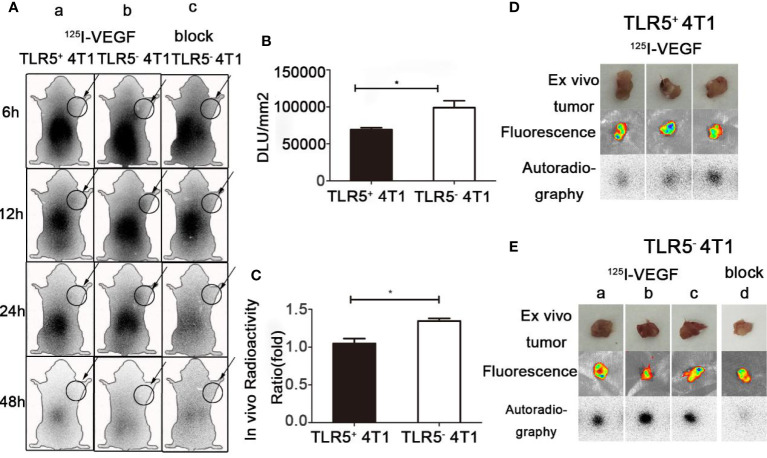
Whole-body phosphor-autoradiography and fluorescence imaging. Whole body phosphor-autoradiography of ^125^I-VEGF in TLR5^+^ 4T1 tumor bearing mice **(A**a**)** and TLR5^−^ 4T1 tumor bearing mice **(A**b**)** at 6, 12, 24, and 48 h post-injection, and whole-body phosphor-autoradiography images of ^125^I-VEGF in TLR5^-^ 4T1 tumor bearing mice with inhibitor blocked group **(A**c**)** at 6, 12, 24, and 48 h post-injection. DLU/mm^2^ measurements in TLR5^+^ and TLR5^‐^4T1 tumors **(B)**. Comparison of *in vivo* radioactivity ratio (the ratio of DLU/mm2 from tumor and opposite muscle of TLR5^+^/^−^ 4T1 tumors in whole-body phosphor-autoradiography at 24 h **(C)**. Phosphor-autoradiography and fluorescence of isolated tumors from TLR5^+^ 4T1 tumor bearing mice **(D)**. Phosphor-autoradiography and fluorescence of isolated tumors from TLR5^−^ 4T1 tumor mice injected with ^125^I-VEGF **(E**a–c**)** and with inhibitor blocked **(E**d**)**. The data were presented from three independent experiments and analyzed by Student’s t test. *P < 0.05.

Whole-body phosphor-autoradiography was performed at 6, 12, 24, and 48 h after injection of the ^125^I-VEGF (**Figure 4**). The uptake of ^125^I-VEGF in tumor increased from 6 h and obviously declined at 48 h. The tumor was clearly visualized at 24 h. Higher radioactivity uptake was detected in 4T1 TLR5^−^ tumor compared with that in 4T1 TLR5^+^ tumors at all checked time points (**Figure 4A**). The semi-quantity of tumor radioactivity in 4T1 TLR5^+^ and 4T1 TLR5^−^ groups was 69329.4 and 99086.8 DLU/mm^2^, respectively (P < 0.05, **Figure 4B**), and T/NT ratio was 1.048 and 1.42586 in 4T1 TLR5^+^ and 4T1 TLR5^−^ groups, respectively (P < 0.05, **Figure 4C**). However, no obvious radioactivity accumulation was found in anti-VEGF mAb blocking group at any checked time point, suggesting that there was a specific accumulation of ^125^I-VEGF in tumor. For *ex vivo* imaging of isolated tumors, TLR5^−^ 4T1 tumors (**Figure 4E**) showed much higher radioactivity accumulation than TLR5^+^ 4T1 tumors (**Figure 4D**), and the fluorescence imaging obviously showed tumor in both groups.

### Biodistribution of ^125^I-anti-TLR5 mAb/^125^I-IgG

**Figure 5:**
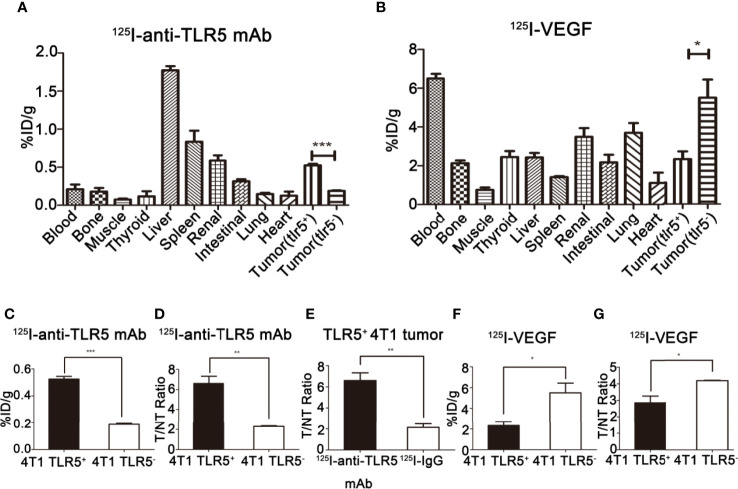
*Ex vivo* biodistribution studies of ^125^I-anti-TLR5 mAb and ^125^I-VEGF. Biodistribution of ^125^I-anti-TLR5 mAb at 48 h post-injection in different organ or tissues **(A)**. Biodistribution of ^125^I-VEGF at 24 h post-injection in different organs or tissues **(B)**. Comparison about the tumor uptake of ^125^I-anti-TLR5 mAb in 4T1 TLR5^+^ and 4T1 TLR5^−^ tumor bearing mice **(C)**. Comparison about T/NT in TLR5^+^/^−^ 4T1 bearing mice injected with ^125^I-anti-TLR5 mAb at 48 h **(D)**. Comparison of tumor uptake in TLR5^+^ 4T1 bearing mice injected with ^125^I-anti-TLR5 mAb and ^125^I-IgG at 48 h **(E)**. Comparison about the tumor uptake of ^125^I-VEGF in 4T1 TLR5^+^ and 4T1 TLR5^−^ tumor bearing mice **(F)**. Comparison about T/NT in TLR5^+^/^−^ 4T1 bearing mice injected with ^125^I-VEGF at 24h **(G)**. The data were presented from three independent experiments and analyzed by Student’s t test. *P < 0.05, **P < 0.01, ***P < 0.001.

The biodistribution of ^125^I-anti-TLR5 mAb/^125^I-IgG was shown in **Figure 5A**. Radioactivity of ^125^I-anti-TLR5 mAb/^125^I-IgG in liver, spleen, and kidney was detected, which suggested that these two radio-probes were mainly metabolized through liver and kidney. The uptake of ^125^I-anti-TLR5 mAb in TLR5^+ ^4T1 tumors was 0.52% ID/g, with T/NT ratio of 6.61 at 48 h post-injection, while in TLR5^− ^4T1 tumors only 0.19%ID/g, with T/NT ratio of 2.30 at the same time point (P < 0.05, **Figures 5C, D** **)**. However, T/NT ratio of ^125^I-IgG was only 2.14 at 48 h in TLR5^+^ tumors (P < 0.01, **Figure 5E**). These results were consistent with the phosphor-autoradiography imaging data.

### Biodistribution of ^125^I-VEGF

The biodistribution of ^125^I-VEGF was shown in **Figure 5B**. The radioactivity in liver, spleen, and kidney was also detected, indicating that the metabolism pathway of ^125^I-VEGF was mainly through the liver and kidney. The %ID/g in TLR5^+^ 4T1 and TLR5^−^ 4T1 tumor was 2.32 and 5.50, respectively (P < 0.05, **Figure 5F**), and T/NT ratio was 2.85 and 4.18, respectively(P < 0.05, **Figure 5G**). The results showed that the uptake of ^125^I-VEGF in TLR5^+^ 4T1 tumor was significantly lower than that in TLR5^−^ 4T1 tumor, which was also consistent with the imaging results.

### H&E and Immunohistochemistry Staining

**Figure 6:**
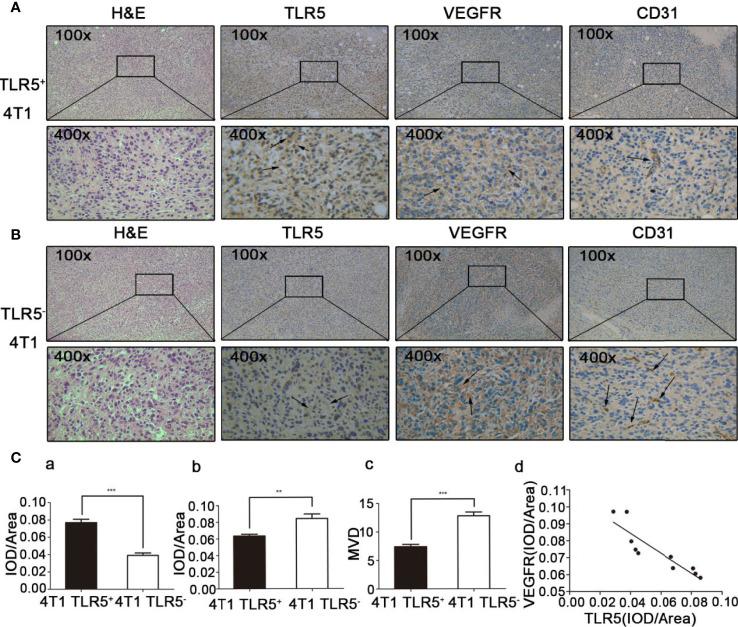
H&E staining and immunohistochemistry staining of ex vivo tumor tissue. TLR5^+^ 4T1 tumors, representative H&E staining, and TLR5/VEGFR/CD31 immunohistochemistry staining at 200× and 400× magnification **(A)**. TLR5^−^ tumors, representative H&E staining, and TLR5/VEGFR/CD31 immunohistochemistry staining at 200× and 400× magnification **(B)**. Statistical analysis of TLR5 detected by IOD/area ratio **(C**a**)**; VEGFR detected by IOD/area ratio **(C**b**)**; MVD count in CD31 labeled tumor **(C**c**)**; and correlation analysis of TLR5 and VEGFR **(C**d**)**. **P < 0.01, ***P < 0.001.

H&E and immunohistochemistry staining results were shown in **Figures 6A, B**. The expression of TLR5 expression was decreased, whereas VEGFR expression increased in TLR5^−^ tumor compared with those in TLR5^+^ tumor. MVD (microvessel density) count in the same slide indicated that there were much more blood vessels in 4T1 TLR5^−^ tumor than that in TLR5^+^ tumor (12.8 vs 7.4; P < 0.0001). There was a negative correlation between TLR5 and VEGFR expression in 4T1 tumors (r^2^ = 0.7972; P < 0.01, **Figure 6C**). These results revealed that down-regulation of TLR5 in TNBC promoted VEGFR expression and angiogenesis, which may have participated in the tumor invasion and metastasis.

## Discussion

TNBC is the most malignant subtype of breast cancer nowadays. Due to the lack of expression of estrogen receptor (ER), progesterone receptor (PR), and epidermal growth factor receptor-2 (EGFR2) (25), there is no effective treatment for TNBC. Chemotherapy is the only systematic treatment for TNBC until now (26), and the treatment effect is not ideal when it is in the advanced stage. Therefore, it is urgent to search for new novel targets for effective diagnosis and therapy of TNBC (4, 26, 27). Angiogenesis, the neovascularization developing from existing blood vessels, is usually in a static state, but it could be promoted in the process of tumor occurrence and development by the increase of angiogenesis factors, such as VEGF and TNF-α, providing a favorable microenvironment for tumor growth, invasion, and metastasis. The tumor cell group and capillary endothelial cell group in the tumor may form a highly integrated ecosystem. Angiogenic factor VEGF, especially VEGF-A, is the most important and key angiogenic factor. VEGF and its receptor are overexpressed in a variety of tumors. It is found that TNBC is potentially sensitive to anti-angiogenic drugs, and its invasiveness is obviously dependent on its angiogenesis (28, 29). The overexpression of VEGFR2 or VEGF-A has been considered as an indicator of poor prognosis in many clinical studies, so intervention toward VEGF and its receptor is an effective measure to prevent tumor growth and development (30, 31). For example, the recent study showed that the highest objective remission rate of PD-L1 antibody combined with VEGF inhibitor in the treatment of TNBC was higher than that of PD-L1 antibody alone (32). VEGF and its receptor VEGFR played an important role in the development of TNBC, and VEGFR was mainly taken as the target and VEGF121, which had a high affinity with VEGFR, seemed to be an ideal tracer (33). It was reported that down-regulation of TLR5 expression could promote proliferation, invasion, and metastasis of TNBC, and TNBC overexpressed VEGF and VEGFR (34). In this study, we confirmed that the expression of VEGFR in 4T1 cells was increased when the expression of TLR5 was down-regulated, suggesting that the down-regulation of TLR5 had an influence on the increase of angiogenesis.

The diagnosis methods of breast cancer include mammography, ultrasound, MRI, and nuclear medicine. However, most of these traditional diagnosis methods have some limitations. For example, mammography cannot distinguish well breast density (4). In dense breast and isodensity masses, the sensitivity and detection rate are low, and it is difficult to distinguish some invasive lobular carcinoma from inflammatory cancer. Ultrasound cannot clearly show small calcification, and it is difficult to diagnose small masses (35). The specificity of MRI is a bit low, and the false-positive rate is high. Nuclear medicine imaging has high sensitivity and can detect small primary lesions and systemic metastases, but the limitation of application lies in the lack of specific imaging agents. Molecular nuclear medicine, which plays an important role in diseases diagnosis, can qualitatively and quantitatively detect physiological and biochemical processes in living tissues, carry out functional metabolism and receptor phenomena dynamically and accurately, and have the advantages of high sensitivity and high detection depth (36). ^18^F-FDG, the most commonly used tracer in clinics, demonstrates certain application value in the early staging of breast cancer. However, ^18^F-FDG is a non-specific imaging agent, and it could result in false positive or false negative misdiagnosis. Inflammation, infection, tuberculosis, and other high metabolic diseases, as well as therapeutic incisions, display high physiological glucose uptake and appear as false-positive diagnosis, whereas some low-grade malignant tumors without high uptake of glucose appear as false-negative misdiagnosis. Therefore, it is necessary to develop molecular probes with high specificity for molecular nuclear imaging. In this study, two radio-probes, ^125^I-anti-TLR5 mAb and ^125^I-VEGF, were prepared to specifically target TLR5 and VEGFR for TNBC imaging. Phosphor-autoradiography and biodistribution study showed that the uptake of ^125^I-anti-TLR5 mAb in TLR5^+^ 4T1 tumor was higher than that in TLR5^−^ 4T1 tumor. However, ^125^I-VEGF uptake in TLR5^+^ 4T1 tumors was much lower than that in TLR5^−^4T1 tumors.

It is undeniable that ^18^F-FDG plays an important role in the staging of TNBC and efficacy evaluation of chemotherapy, but the specificity of ^18^F-FDG is not high (37). In this study, comparative experiment of ^125^I-IgG and ^125^I-anti-TLR5 mAb imaging, as well as ^125^I-VEGF and ^125^I-VEGF blocking imaging, revealed the specificity of ^125^I-anti-TLR5 mAb and ^125^I-VEGF, and both of ^125^I-anti-TLR5 mAb and ^125^I-VEGF were much more specific than ^18^F-FDG. Moreover, the accumulation of ^125^I-anti-TLR5 mAb and ^125^I-VEGF in lung and heart was quite low, reducing the background interference. These two radio-probes were much more conducive for targeted imaging of tumors, which made it possible to verify the effect of down-regulation of TLR5 in TNBC on VEGFR by *in vivo* autoradiography and non-invasive visualization. However, there are some shortcomings in this study, such as the lack of *in vitro* experiments to verify the effect of down-regulation of TLR5 on angiogenesis. Therefore, whether the effect of down-regulation of TLR5 on the biological behavior of TNBC is through affecting VEGFR and angiogenesis still needs further investigation.

## Conclusions

In conclusion, we found that the expression of TLR5 was in negative correlation with the expression of VEGFR in TNBC. The down-regulation of TLR5 in TNBC may promote the VEGFR expression and angiogenesis, resulting in the proliferation of TNBC cells. The effect of TLR5 on the biological behavior of TNBC may be through the regulation of VEGFR expression and angiogenesis, and TLR5/VEGF might be a better indicator for monitoring the development of TNBC.

## Data Availability Statement

The raw data supporting the conclusions of this article will be made available by the authors, without undue reservation.

## Ethics Statement

The animal study was reviewed and approved by the Animal Care and Use Committee of Shandong University with the corresponding ethical approval code (LL-201602040, 2016-2022).

## Author Contributions

Conceptualization, FG and GH. Methodology, GH. Validation, WJ, YH, TL, and CZ. Formal analysis, FG. Investigation, WJ, YH, TL, and CZ. Data curation, WJ, YH, TL, and CZ. Writing—original draft preparation, WJ, FG, and GH. Writing—review and editing, YH, TL, and CZ. Supervision, FG and GH. Project administration, WJ, YH, TL, and CZ. Funding acquisition, FG and GH. All authors contributed to the article and approved the submitted version.

## Funding

This research was funded by grants from the National Natural Science Foundation of China (81371601) and Natural Science Foundation of Shandong Province (ZR2019MH019, ZR2019BA015).

## Conflict of Interest

The authors declare that the research was conducted in the absence of any commercial or financial relationships that could be construed as a potential conflict of interest.

## References

[1] Foulkes WD Smith IE Reis JS. Triple-Negative Breast Cancer. New Engl J Med (2010) 363:1938–48. 10.1056/NEJMra1001389 21067385

[2] Pei X Wang X Xian J Mi J Gao J Li X. Metformin and Oxyphotodynamic Therapy as a Novel Treatment Approach for Triple-Negative Breast Cancer. Ann Transl Med (2020) 8(18):1138. 10.21037/atm-20-5704 33240987 PMC7576064

[3] Shao F Sun H Deng CX. Potential Therapeutic Targets of Triple-Negative Breast Cancer Based on Its Intrinsic Subtype. Oncotarget (2017) 8:73329–44. 10.18632/oncotarget.20274 PMC5641215 29069872

[4] Jhan JR Andrechek ER. Triple-Negative Breast Cancer and the Potential for Targeted Therapy. Pharmacogenomics (2017) 18:1595–609. 10.2217/pgs-2017-0117 PMC5694022 29095114

[5] Zhu X Zhou W. The Emerging Regulation of veGFR-2 in Triple-Negative Breast Cancer. Front Endocrinol (2015) 6:159. 10.3389/fendo.2015.00159 PMC4598588 26500608

[6] Jansson S Bendahl P-O Grabau DA Falck A-K Fernö M Aaltonen K. The Three Receptor Tyrosine Kinases c-KIT, VEGFR2 and PDGFR Alpha, Closely Spaced at 4q12, Show Increased Protein Expression in Triple-Negative Breast Cancer. PloS One (2014) 9:e102176. 10.1371/journal.pone.0102176 25025175 PMC4098911

[7] Gao ZY Shi M Wang Y Chen J Ou Y. Apatinib Enhanced Anti-Tumor Activity of Cisplatin on Triple-Negative Breast Cancer Through Inhibition of VEGFR-2. Pathol Res Pract (2019) 215:152422. 10.1016/j.prp.2019.04.014 31079851

[8] Malekian S Rahmati M Sari S Kazemimanesh M Kheirbakhsh R Muhammadnejad A. Expression of Diverse Angiogenesis Factor in Different Stages of the 4T1 Tumor as a Mouse Model of Triple-Negative Breast Cancer. Adv Pharm Bull (2020) 10:323–8. 10.34172/apb.2020.039 PMC7191227 32373503

[9] Mohammed RAA Green A El-Sheikh S Paish EC Ellis IO Martni SG. Prognostic Significance of Vascular Endothelial Cell Growth Factor (VEGF) -A, -C and -D in Breast Cancer and Their Relationship With Angio- and Lymphangiogenesis. Ejc Suppl (2007) 5:28–8. 10.1016/S1359-6349(07)71780-9 PMC2360132 17353919

[10] Mohammed RAA Green A El-Sheikh S Paish EC Ellis IO Martni SG. Prognostic Significance of Vascular Endothelial Cell Growth Factors -A, -C and -D in Breast Cancer and Their Relationship With Angio- and Lymphangiogenesis. Brit J Cancer (2007) 96:1092–100. 10.1038/sj.bjc.6603678 PMC2360132 17353919

[11] Bender RJ Mac Gabhann F. Expression of VEGF and Semaphorin Genes Define Subgroups of Triple Negative Breast Cancer. PloS One (2013) 8:e61788. 10.1371/journal.pone.0061788 23667446 PMC3648524

[12] Hnatowich DJ. Observations on the Role of Nuclear Medicine in Molecular Imaging. J Cell Biochem (2002) 87:18–24. 10.1002/jcb.10400 12552598

[13] Czernin J Sonni I Razmaria A Calais J. The Future of Nuclear Medicine as an Independent Specialty. J Nucl Med (2019) 60:3S–12S. 10.2967/jnumed.118.220558 31481589

[14] Ueda M. Development of Radiolabeled Molecular Imaging Probes for *In Vivo* Analysis of Biological Function. Yakugaku Zasshi (2016) 136:659–68. 10.1248/yakushi.15-00279 27040347

[15] Govaert GAM Glaudemans AWJM. Nuclear Medicine Imaging of Posttraumatic Osteomyelitis. Eur J Trauma Emerg S (2016) 42:397–410. 10.1007/s00068-016-0647-8 PMC4969346 26886235

[16] Langbein T Weber WA Eiber M. Future of Theranostics: An Outlook on Precision Oncology in Nuclear Medicine. J Nucl Med (2019) 60:13s–9s. 10.2967/jnumed.118.220566 31481583

[17] Taieb D Hicks RJ Hindié E Guillet BA Avram A Ghedini P. European Association of Nuclear Medicine Practice Guideline/Society of Nuclear Medicine and Molecular Imaging Procedure Standard 2019 for Radionuclide Imaging of Phaeochromocytoma and Paraganglioma. Eur J Nucl Med Mol I (2019) 46:2112–37. 10.1007/s00259-019-04435-z PMC7446938 31254038

[18] Singnurkar A Poon R Metser U. Comparison of 18F-FDG-PET/CT and 18F-FDG-PET/MR Imaging in Oncology: A Systematic Review. Ann Nucl Med (2017) 31:366–78. 10.1007/s12149-017-1164-5 28353197

[19] Kaczanowska S Joseph AM Davila E. TLR Agonists: Our Best Frenemy in Cancer Immunotherapy. J Leukocyte Biol (2013) 93:847–63. 10.1189/jlb.1012501 PMC3656332 23475577

[20] Palestro CJ Love C. Nuclear Medicine Imaging in Fever of Unknown Origin: The New Paradigm. Curr Pharm Design (2018) 24:814–20. 10.2174/1381612824666171129194507 29189130

[21] Shi D Zhao S Jiang W Zhang C Liang T Hou G. TLR5: A Prognostic and Monitoring Indicator for Triple-Negative Breast Cancer. Cell Death Dis (2019) 10:954. 10.1038/s41419-019-2187-8 31852883 PMC6920449

[22] Kau P Nagaraja GM Zheng H Gizachew D Galukande M Krishnan S. A Mouse Model for Triple-Negative Breast Cancer Tumor-Initiating Cells (TNBC-TICs) Exhibits Similar Aggressive Phenotype to the Human Disease. BMC Cancer (2012) 12:120. 10.1186/1471-2407-12-120 22452810 PMC3340297

[23] Shi D Hou G. TLR5 Is a New Reporter for Triple-Negative Breast Cancer Indicated by Radioimmunoimaging and Fluorescent Staining. Eur J Immunol (2019) 49:1996–6. 10.1111/jcmm.14707 PMC6850942 31576678

[24] Liu W Zhang C Cao H Shi D Zhao S Liang T. Radioimmunoimaging of I-125-Labeled Anti-CD93 Monoclonal Antibodies in a Xenograft Model of Non-Small Cell Lung Cancer. Oncol Lett (2019) 18:6413–22. 10.3892/ol.2019.11036 PMC6896371 31819775

[25] Seal MD Chia SK. What Is the Difference Between Triple-Negative and Basal Breast Cancers? Cancer J (2010) 16:12–6. 10.1097/PPO.0b013e3181cf04be 20164685

[26] Nedeljkovic M Damjanovic A. Mechanisms of Chemotherapy Resistance in Triple-Negative Breast Cancer-How We Can Rise to the Challenge. Cells-Basel (2019) 8:957. 10.3390/cells8090957 PMC6770896 31443516

[27] Vagia E Mahalingam D Cristofanilli M. The Landscape of Targeted Therapies in TNBC. Cancers (2020) 12:916. 10.3390/cancers12040916 PMC7226210 32276534

[28] Mauro CD Rosa R D 'Amato V Ciciola P Servetto A Marciano R. Hedgehog Signalling Pathway Orchestrates Angiogenesis in Triple-Negative Breast Cancers. Brit J Cancer (2017) 116:1425–35. 10.1038/bjc.2017.116 PMC5520095 28441382

[29] Hatem R Labiod D Chteau-Joubert S Plater LD Botty RE Vacher S. Vandetanib as a Potential New Treatment for Estrogen Receptor-Negative Breast Cancers. Int J Cancer (2016) 138:2510–21. 10.1002/ijc.29974 26686064

[30] Siveen KS Prabhu K Krishnankutty R Kuttikrishnan S Tsakou M Alali FQ. Vascular Endothelial Growth Factor (VEGF) Signaling in Tumour Vascularization: Potential and Challenges. Curr Vasc Pharmacol (2017) 15:339–51. 10.2174/1570161115666170105124038 28056756

[31] Taurone S Galli F Signore A Agostinelli E Dierckx RA Minni A. VEGF in Nuclear Medicine: Clinical Application in Cancer and Future Perspectives (Review). Int J Oncol (2016) 49:1766–6. 10.3892/ijo.2016.3636 27277340

[32] Wu F Xu P Chow A Man S Krüger J Khan KA. Pre- and Post-Operative Anti-PD-L1 Plus Anti-Angiogenic Therapies in Mouse Breast or Renal Cancer Models of Micro- or Macro-Metastatic Disease. Brit J Cancer (2019) 120:196–206. 10.1038/s41416-018-0297-1 30498230 PMC6342972

[33] Cai W Chen X. Multimodality Imaging of Vascular Endothelial Growth Factor and Vascular Endothelial Growth Factor Receptor Expression. Front Biosci-Landmrk (2007) 12:4267–79. 10.2741/2386 17485373

[34] Chen S Yuan W Fu Z Huang Y Yang J Xue J. Toll-Like Receptor 5 Gene Polymorphism Is Associated With Breast Cancer Susceptibility. Oncotarget (2017) 8:88622–9. 10.18632/oncotarget.20242 PMC5687632 29179462

[35] Hudis CA Gianni L. Triple-Negative Breast Cancer: An Unmet Medical Need. Oncologist (2011) 16:1–11. 10.1634/theoncologist.2011-S1-01 21278435

[36] Abou DS Pickett JE Thorek DLJ. Nuclear Molecular Imaging With Nanoparticles: Radiochemistry, Applications and Translation. Brit J Radiol (2015) 88:20150185. 10.1259/bjr.20150185 26133075 PMC4730968

[37] Raccagni I Belloli S Valtorta S Stefano A Presotto L Pascali C. [F-18]FDG and [F-18]FLT PET for the Evaluation of Response to Neo-Adjuvant Chemotherapy in a Model of Triple Negative Breast Cancer. PloS One (2018) 13:e0197754. 10.1371/journal.pone.0197754 29791503 PMC5965848

